# Description of a new species of the genus *Tribasodites* Jeannel (Coleoptera, Staphylinidae, Pselaphinae) from East China with a key to world species

**DOI:** 10.3897/zookeys.64.454

**Published:** 2010-10-22

**Authors:** Mei-Jun Zhao, Zi-Wei Yin, Li-Zhen Li

**Affiliations:** Department of Biology, College of Life and Environmental Sciences, Shanghai Normal University, Shanghai, P. R. China

**Keywords:** Coleoptera, Staphylinidae, Pselaphinae, Tribasodites, new species, East China, taxonomy

## Abstract

A remarkable new species of the genus Tribasodites, 1960, Tribasodites spinacaritus **sp. n.** is described and illustrated from Zhejiang Province, East China. A key to world species of the genus is provided. Systematic position of the new taxon is discussed.

## Introduction

The genus Tribasodites was erected by Jeannel (1960) to accommodate two new species, Tribasodites antennalis and Tribasodites frontalis, both described from North India. Twenty-six years later, Nomura (1986) added a third species to the genus, Tribasodites picticornis collected in a colony of Paratrechina flavipes (Smith) in Japan. Afterwards, Nomura (2000) listed eight species of Tribasodites based on the materials collected from Yunnan, but without specific name and description. Then, (Nomura (2007a, 2007b) transferred two species Batrisodes semipunctatus Raffray, 1912 (Taiwan) and Batrisodes coiffaiti Jeannel, 1958 (Japan) to Tribasodites. So far, five species of the genus have been known in the world.

The genus Tribasodites can be readily distinguished from its allies by a combination of the following characters: 1) male with sexually modified head or antenna; 2) pronotum with a pair of spines or denticles on lateral sides, disc with a median longitudinal sulcus; 3) elytra each with three basal foveae; 4) male metatrochanter spinulate or simple; 5) the first visible tergite (morphologically tergite IV) weakly concave near base, paratergites reduced to a pair of triangular plates demarcated by lateral carinae; 6) aedeagus asymmetrical, usually with a dorsal apophysis well-developed to totally reduced or absent.

During studies on the Chinese Tribasodites, some specimens were collected during a short expedition to Tiantongshan Mountain, Zhejiang Province, East China. The examination of the material revealed a remarkable species which is unknown to science.

The purpose of the present paper is to describe this new species under the name of Tribasodites spinacaritus sp. n., and to provide a key to all known species. The systematic position of the new species is also discussed.

## Material and methods

Specimens were collected from decaying leaf litter of the forest floor by sifting and were killed with ethyl acetate and then dried. Dissections were made in 75% ethanol; genitalia and small parts were mounted in Euparal on plastic slides that were placed on the same pin with the specimens. Photos of habitus were taken by a Canon EOS 40D Camera mounted with an MP-E 65 mm Macro Photo Lens; photos of dissected parts were taken by a Canon G9 camera mounted on an Olympus CX21 microscope; line drawings were made by Adobe Illustrator CS2.

The terminology follows Chandler, 2001. ‘/’ slash is used in the text to separate different lines of the label.

Type series are deposited in the Insect Collection of Shanghai Normal University, Shanghai, China (=SHNUC)

## Taxonomy

### Tribasodites spinacaritus

Yin, Li & Zhao sp. n.

urn:lsid:zoobank.org:act:ECD13679-7279-4023-9CBF-6C3E353F8EB2

Figs 1 [Fig F2] 22 

#### Type locality.

East China, Zhejiang Province, Tiantongshan Mountain.

#### Type material.

HOLOTYPE, male: ‘CHINA: ZHEJIANG Prov. / Ning’bo City / Tiantongshan Mt./alt. 350 m, 24–26.iv.2009/Ting FENG leg.’ (SHNU). PARATYPES: 4 males, 6 females, same label data as holotype (SHNU)

**Figures 1–2.**
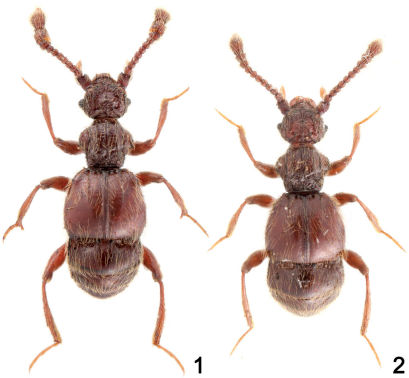
Dorsal habitus of *Tribasodites spinacaritus* sp. n. **1** male **2** female.

#### Description.

##### Male.

Length 2.2–2.4 mm (Fig. 1). Reddish brown, maxillary palpi and tarsi lighter.

**Figures 3–14. F2:**
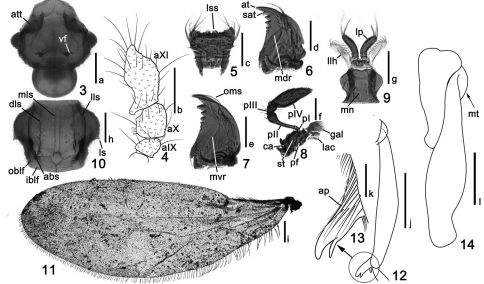
Details of *Tribasodites spinacaritus* sp. n. **3** head **4** male antennal club **5** labrum **6** right mandible, dorsal view **7** left mandible, ventral view **8** left maxilla **9** labium **10** pronotum **11** left metathoracic wing **12** mesotibia **13** apical protuberance of mesotibia, enlarged **14** metatrochanter and metafemur. Scales: **a**, **b**, **h**, **i**, **j** and **l** = 0.2 mm, **c**, **d**, **e**, **f**, **g**, and **k** = 0.1 mm. Abbreviations: **abs** = antebasal spine; **ap** = apical protuberance; **at** = apical tooth; **att** = antennal tubercle; **aIX–aXI** = antennomere **IX**–antennomere XI; **ca** = cardo; **dls** = disc longitudinal sulcus; **gal** = galea; **iblf** = inner basolateral foveae; **lac** = lacinia; **llh** = lateral lobe of hypopharynx; **lls** = lateral longitudinal sulcus; **lp** = labial palpus; **ls** = lateral spine; **lss** = labral specialized setae; **mdr** = mandibular dorsal ridge; **mls** = median longitudinal sulcus; **mn** = mentum; **mt**= metatrochanter; **mvr** = mandibular ventral ridge; **oblf** = outer basolateral foveae; **oms** = outer marginal seta; **pf** = palpifer; **pI–pIV** = palpomere I–palpomere IV; **rmt** = row of median teeth; **sat** = subapical tooth; **st** = stipes.

Head (Fig. 3) slightly wider than long, nearly triangular, covered with short hair on dorsal surface. Clypeus arcuate on anterior margin. Labrum (Fig. 5) longer than wide, with rows of long setae anterolaterally, anteromedian margin with four minute specialized setae. Mandible (Figs 6–7) with one large apical tooth and much smaller subapical tooth and row of median teeth on cutting edge; outer margin with long seta in apical one-third. Maxillary palpus (Fig. 8) with palpomere I minute, II pedunculate with anterior third broadened, III nearly triangular, IV predominately large, nearly fusiform. Labium (Fig. 9) slightly wider than long, rounded laterally, labial palpus composed of large basal segment and setae-like terminal segments; lateral lobe setose. Frons depressed between antennal tubercles. Vertex convex, with one pair of vertexal foveae connected by short U-shaped carina and with median keel. Eyes large and prominent, situated in basal two-fifths of head length, not emarginated, multifaceted, each composed of about 55 facets. Postgenae nearly rounded, with pair of lateral carinae extended to antennal tubercles. Gular area slightly depressed; gular foveae merged into single pit. Gular carina present. Antenna long and elongate, scape large, about 1.5 times as long as wide. Pedicle much smaller than scape, subcylindrical; antennomeres III–VIII each wider than long, transverse; club three-segmented with antennomeres IX–XI (Fig. 4) modified, roughly granulated. X about twice as wide as and 1.5 times as long as VIII, nearly triangular, X slightly longer than wide, inner side strongly concaved, with several short and thick setae; XI the largest, widest in the middle, inner antebasal part strongly protuberant.

Pronotum (Fig. 10) wider than long, lateral sides each with one median spine; with one pair of lateral and one pair of discal longitudinal sulci, one pair of antebasal spines near basal margin of pronotum, one pair of lateral antebasal foveae and two pairs of basolateral foveae distinct.

Elytra (Fig. 17) convex, longer than wide, narrowed toward base. Each tri-foveate; discal stria extended to half of elytral length; sutural stria present. Metathoracic wings (Fig. 11) fully developed, widest at middle, gradually narrowed from middle toward apex and base, apex rounded. Venter with clear pairs of lateral mesoventral foveae and lateral metaventral foveae.

Legs normal in structure. Mesotibia (Figs 12–13) with apical protuberance. Metatrochanter (Fig. 14) not spinose.

**Figures 15–22.**
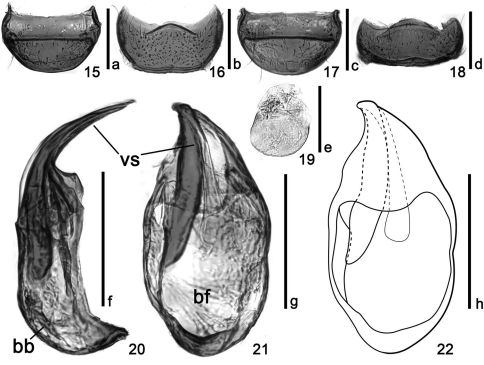
Details of *Tribasodites spinacaritus* sp. n. **15** male tergite VIII **16** male sternite VIII **17** femal tergite VIII **18** female sternite VIII **19** male sternite IX **20** aedeagus, lateral view **21–22** aedeagus in ventral view. Scales: **a**, **b**, **c**, **d**, **f**, **g**, and **h** = 0.2 mm; **e** = 0.1 mm. Abbreviations: **bb** = basal bulb; **bf** = basal foramen; **vs** = ventral stalk.

Abdomen with first visible tergite (morphologically tergite IV) largest, mediobasal foveae, basolateral foveae and basomedian cavity present; discal carinae very short; tergites V–VII successively shorter and narrower, each with pair of lateral foveae. Tergite VIII (Fig. 15) transverse, posterior side nearly flattened. Sternites IV–VII each transverse, successively shorter and narrower, each with pair of lateral foveae. Sternite VIII (Fig. 16) transverse, with anterior margin strongly emarginated and posterior margin flattened. Sternite IX (Fig. 18) membranous.

Aedeagus (Figs 20–22) with dorsal apophysis totally absent; parameres reduced, forming a ventral stalk with median lobe; endophallus elongate, very weakly sclerotized, gradually expanded posteriad; basal foramen large; basal bulb round posteriorly.

##### Female

Body size similar to male (Fig. 2). Antennal club not modified. Eyes smaller than in male. Metathoracic wing slightly smaller than in male. Mesotibiae not protuberant at apex. Tergite VIII (Fig. 17) semispheric. Sternite VIII (Fig. 18) transverse. Sternite IX reduced.

#### Etymology.

The specific name refers to the metatrochanter without any spine or protuberance.

#### Relationship.

The male genitalia of the new species is somewhat similar to that of Batrisodes or some species of Batrisus genus-group, and the male spine on the hind trochanter is absent in the new species, which makes the new species looks similar to Batrisodes in some male sexual characters. But it is still quite different from Batrisodes. The new species is placed in Tribasodites because of the following reasons: 1) the prothorax of the new species has basic characters (spinulate lateral margins) of the Tribasodes genus-group, which never occurs in the Batrisodes belonging to Batrisus genus-group (both genus-groups were defined by Nomura and Idris 2003), 2) its male genitalia is strictly asymmetrical, which does not match the symmetrical male genitalia of Batrisodes.

The new species is most close related to Tribasodites picticornis and Tribasodites antennalis by relatively large body size and sexually modified antennal club. Tribasodites spinacaritus can be readily distinguished by the absence of metatrochanteral spine on posterior margin and simple structure of aedeagus, while all the other species have spinulate metatrochanter and aedeagus with fully-developed dorsal apophysis.

#### Key to species of Tribasodites Jeannel

**Table:** 

1	Male sexual character presents only on head, head with large excavation on vertex in male	2
–	Male sexual character present only on antenna	3
2	Body medium-sized, less than 2.0 mm in length; head with a short median keel and a pair of acinous patches above postgenae	Tribasodites semipunctatus (Raffray, 1912) (China: Taiwan)
–	Body large-sized, no less than 3.0 mm in length; head lacking median keel and pair of acinous patches above postgenae	Tribasodites frontalis Jeannel, 1960 (India: Himachal Pradesh, Uttar Pradesh)
3	Metatrochanter without spine or protuberance on posterior margin	Tribasodites spinacaritusYin et al., sp. n. (China: Zhejiang)
–	Metatrochanter with a spine or a protuberance on posterior margin	4
4	Body large-sized, no less than 2.5 mm in length; antennomere X nearly triangular, not modified in structure	Tribasodites antennalis Jeannel, 1960 (India: Himachal Pradesh, Uttar Pradesh)
–	Body medium-sized, less than 2.0 mm in length; antennomere X not triangular, variously modified in structure	5
5	Eyes very small, less than 40 facets; antennomere IX clearly larger than X in male, with a conical protuberance on inner side, slightly smaller than X and symmetrical in female; pronotum without lateral process, but with a pair of small antebasal denticles; each protibia with a large denticle on inner side near the middle in male	Tribasodites coiffaiti (Jeannel, 1958) (Japan: Kawauchi)
–	Eyes developed, more than 50 facets; antennomere IX smaller than X in male, X asymmetrical in male, symmetrical and subglobose in female; pronotum with a pair of large lateral processes and with a pair of antebasal denticles; protibia slender and simple in both sexes	Tribasodites picticornis Nomura, 1986 (Japan: Okinawa Island)

## Supplementary Material

XML Treatment for Tribasodites spinacaritus
